# Structure of the Response Regulator NsrR from *Streptococcus agalactiae*, Which Is Involved in Lantibiotic Resistance

**DOI:** 10.1371/journal.pone.0149903

**Published:** 2016-03-01

**Authors:** Sakshi Khosa, Astrid Hoeppner, Holger Gohlke, Lutz Schmitt, Sander H. J. Smits

**Affiliations:** 1 Institute of Biochemistry, Heinrich Heine University Duesseldorf, Universitaetsstr. 1, 40225, Duesseldorf, Germany; 2 X-Ray Facility and Crystal Farm, Heinrich Heine University Duesseldorf, Universitaetsstr. 1, 40225, Duesseldorf, Germany; 3 Institute of Pharmaceutical and Medicinal Chemistry, Heinrich Heine University Duesseldorf, Universitaetsstr. 1, 40225, Duesseldorf, Germany; Centre National de la Recherche Scientifique, Aix-Marseille Université, FRANCE

## Abstract

Lantibiotics are antimicrobial peptides produced by Gram-positive bacteria. Interestingly, several clinically relevant and human pathogenic strains are inherently resistant towards lantibiotics. The expression of the genes responsible for lantibiotic resistance is regulated by a specific two-component system consisting of a histidine kinase and a response regulator. Here, we focused on a response regulator involved in lantibiotic resistance, NsrR from *Streptococcus agalactiae*, and determined the crystal structures of its N-terminal receiver domain and C-terminal DNA-binding effector domain. The C-terminal domain exhibits a fold that classifies NsrR as a member of the OmpR/PhoB subfamily of regulators. Amino acids involved in phosphorylation, dimerization, and DNA-binding were identified and demonstrated to be conserved in lantibiotic resistance regulators. Finally, a model of the full-length NsrR in the active and inactive state provides insights into protein dimerization and DNA-binding.

## Introduction

The dramatic rise in antibiotic resistance has posed a major threat to the treatment of infectious diseases. This has led to the search for novel antibiotics that can be used as pharmaceuticals against human pathogenic bacteria. One of the potential antibiotic alternatives are lantibiotics [1]. Lantibiotics are small antimicrobial peptides (30–50 amino acids in length), which are produced by several Gram-positive bacterial strains. They are post-translationally modified and contain specific lanthionine/methyl-lanthionine rings, which are crucial for their high antimicrobial activity [2]. Lantibiotics are for example highly effective against various Gram-positive, human pathogenic bacteria including *Streptococcus pneumoniae* and several methicillin-resistant *Staphylococcus aureus* (MRSA) strains [3]. The high potency of lantibiotics for medical usage has already been noticed, and several lantibiotics are already included in clinical trials [4,5]. Their high potency is highlighted by the fact that, although being extensively used in food industry, resistance has not been described so far [6]. Nisin is the most prominent member of the lantibiotic family and is able to inhibit cell growth, penetrates the membranes of various Gram-positive bacteria, and is characterized by five specific (methyl-)lanthionine rings, which are crucial for stability and activity in the nanomolar range [7,8]. Thus, the lantibiotic producer strains have an inbuilt self-protection mechanism (immunity) to prevent cell death caused due to the action of its cognate lantibiotic [9]. This immunity system consists of a membrane–associated lipoprotein (usually referred to as LanI) and/or an ABC transporter (termed as LanFEG and comprising three subunits) [10]. Although some lantibiotics such as Pep5, epicidin, epilancin, and lactocin S only require LanI for immunity, other lantibiotics with a dual mode of action involving pore formation and lipid II binding such as nisin, subtilin, epidermin, gallidermin, and lacticin 3147 require additionally the presence of LanFEG [11–13]. Examples for LanFEG are NisI and NisFEG of the nisin system, SpaI and SpaFEG conferring immunity towards subtilin, and PepI constituting the immunity system of Pep5 producing strains [14]. Structural data are reported for the immunity proteins NisI from *Lactococcus lactis* [15], SpaI from *Bacillus subtilis* [16] and MlbQ from the lantibiotic NAI-107 producer strain *Microbispora* ATCC PTA-5024 [17].

Recently, gene clusters were identified in certain clinically relevant human pathogenic strains such as *Streptococcus agalactiae*, *S*. *aureus*, and others that confer inherent resistance against specific lantibiotics such as nisin [18–20] and resemble the genetic architecture of the lantibiotic immunity genes found in the producing strains. Within these resistance operons, genes encoding for a membrane-associated protease and an ABC transporter were identified. Expression of these proteins provides resistance against lantibiotics. Recently, the structure of *Sa*NSR from *S*. *agalactiae* was solved which provides resistance against nisin by a protease activity [21]. Furthermore, the upregulation of these genes is mediated by a specific two-component system (TCS) similar to the one found in lantibiotic producing strains [9,18], consisting of a sensor histidine kinase (HK) and a response regulator (RR), apparently mediate the expression of the resistance proteins [22]: HK senses the external lantibiotic and, upon receiving the stimuli, auto-phosphorylates at a conserved histidine residue within the cytosol; this high-energetic phosphoryl group is then transferred to the associated RR inducing a conformational change there, which activates the RR to evoke the cellular response. Bacteria have the ability to sense and survive various environmental stimuli through adaptive responses, which are regulated by TCSs [23]. These processes include drug resistance, quorum-sensing, phosphate uptake, sporulation, and osmoregulation [24]. The absence of TCSs within mammals makes them unique targets for novel antimicrobial drugs [25].

The expression of the lantibiotic-resistance genes via TCS is generally regulated by microorganism-specific lantibiotics, which act via external stimuli. Some examples of TCS are: BraRS in *S*. *aureus* which is induced by bacitracin, nisin and nukacin-ISK-1 resistance [26], BceRS in *Bacillus spp*. that is induced by actagardine and mersacidin resistance [27], LcrRS in *Streptococcus mutans* induced by nukacin-ISK-1 and lacticin 481 [19] and LisRK of *Listeria monocytogenes* induced by nisin resistance [28]. Furthermore, multiple lantibiotics can induce the TCS CprRK from *Clostridium difficile*, leading to the expression of the genes localized on the *cpr* operon, resulting in resistance against several lantibiotics of which nisin, gallidermin, subtilin, and mutacin 1140 are some examples. Interestingly, the histidine kinase contains two-transmembrane helices but lacks an extracellular sensory domain, and are therefore known as ‘intramembrane-sensing’ histidine kinases [29,30]. It has been suggested that in addition to conferring general resistance against lantibiotics, the BceAB-type transporters assist in signalling as via the presence of a large extracellular domain within the transmembrane segment indicated by experimental evidence from various systems [20,26,31–33].

The recently discovered *nsr* gene cluster of the human pathogen *S*. *agalactiae* encodes for the resistance protein NSR and the ABC transporter NsrFP, both conferring resistance against nisin [18]. Homologous operons have been identified in various human pathogenic strains such as *Staphylococcus epidermis* and *Streptococcus ictaluri* based on the high sequence identity of NSR and NsrFP. In this gene cluster, the TCS NsrRK is responsible for the expression of the *nsr* and *nsrFP* genes [18]. The similarity of the TCS within all the described nisin resistance operons suggests an expression specifically induced by nisin [18]. Thus, NsrRK might be a useful target to combat inherently pathogenic lantibiotic-resistant strains.

Generally, RRs consist of two distinct structural domains, a receiver domain (RD) and an effector domain (ED), that are separated from each other by a flexible linker. RDs contain a highly conserved aspartate residue, which acts as a phosphoryl acceptor that becomes phosphorylated by the kinase domain of the histidine kinase upon reception of an external signal. The ED is thereby activated and binds to the designated promoters, thus initiating transcription of the target genes.

The RRs are classified into different subfamilies depending on the three-dimensional structure of their EDs [24,34]. The OmpR/PhoB subfamily is the largest subgroup of RRs and comprises approximately 40% of all response regulators in bacteria. All their members are characterized by a winged helix-turn-helix (wHTH) motif [35]. Although numerous structures of the single domains are known, only a few structures of full-length OmpR/PhoB-type RRs have been determined: RegX3 (PDB code: 2OQR) [36], MtrA (PDB code: 2GWR) [37], PrrA (PDB code: 1YS6) [38] and PhoP (PDB code: 3R0J) [39] from *Mycobacterium tuberculosis*; DrrB (PDB code: 1P2F) [40] and DrrD (PDB code: 1KGS) [41] from *Thermotoga maritima*; and KdpE from *Escherichia coli* (PDB code: 4KNY) [42]. The various structures of RRs reveal that in addition to being in either “inactive” or “active” state, the RRs can also exist in two distinct conformations: “open” and “closed”. MtrA and PrrA exhibit a very compact, closed structure with the DNA-binding sequence, called recognition helix, of the ED being inaccessible to DNA [37,38]. The structures of DrrD and DrrB exist in an open conformation, here the recognition helix is fully exposed [40,41], suggesting that RRs are flexible in solution and can adopt multiple conformations.

Here, we describe the crystal structures of the N-terminal RD and the C-terminal ED of the lantibiotic resistance-associated RR NsrR from *S*. *agalactiae*. NsrR is part of the nisin resistance operon [18]. The expression of the genes of this operon is induced by a TCS consisting of the HK NsrK and the RR NsrR. Based on the crystal structures of both the domains, modeling was employed to shed light on the putative DNA-bound state of full-length NsrR.

## Materials and Methods

### Cloning, expression and purification NsrR

NsrR was constructed, expressed, and purified as described previously [43]. In brief, the *nsrR* gene (accession no. HG939456.1) from *S*. *agalactiae* COH1 was ligated into the expression vector pET24a allowing expression in *E*. *coli* with a His_6_-tag introduced at the C-terminus. The resulting plasmid pET24a-NsrR was transformed into *E*. *coli* BL21 (*DE3*) for expression. A single transformed colony was inoculated into 20 ml LB media containing 30 μg/ml kanamycin. The culture was grown for 14 h at 310 K with shaking at 200 rpm. 4 l LB media with 30 μg/ml kanamycin were inoculated with the overnight culture at an OD_600_ of 0.05 and grown at 310 K with shaking at 170 rpm until an OD_600_ of 0.3 was reached. Subsequently, temperature was lowered to 291 K, and cells were further grown until an OD_600_ of 0.8 was reached before inducing the expression by addition of 1 mM IPTG. Cells were further grown for 15 h and harvested by centrifugation at 8000 rpm for 20 min at 277 K. The harvested cell pellet was re-suspended in 10 ml of buffer *A* (50 mM Tris pH 8.0, 50 mM NaCl, 2 mM PMSF and 10% (v/v) glycerol) and 10 mg of DNase (Deoxyribonuclease I from bovine pancreas, Sigma Aldrich) was added. Cells were lysed using a cell disruptor (Constant Cell Disruption Systems, United Kingdom) at 2.6 × 10^5^ kPa. The lysate was centrifuged at 42000 rpm for 60 min using a Ti60 rotor to remove non-lysed cells and cell debris.

20 mM imidazole was added to the cleared lysate prior to applying it onto a Ni^2+^ loaded Hi-Trap HP Chelating column (GE Healthcare) pre-equilibrated with buffer *B* (20 mM Tris pH 8.0, 250 mM NaCl and 20 mM imidazole, 2 mM PMSF). The column was washed with six column volumes of buffer *B*. Protein was eluted with a linear gradient of imidazole from 20 mM to 400 mM in buffer *B*. The fractions containing NsrR were pooled and concentrated up to 8 mg/ml in an Amicon centrifugal filter concentrator with a 10 kDa cut-off membrane (Millipore). The concentrated protein was further purified by size exclusion chromatography using a Superdex 200 GL 10/300 column (GE Healthcare), equilibrated with buffer *C* (25 mM Tris pH 9.0, 50 mM NaCl, 2 mM PMSF). The eluted protein fractions were pooled and concentrated to 11 mg/ml as described above. The purity of the protein was analyzed with 15% SDS-PAGE using colloidal Coomassie blue staining [44].

### Crystallization of NsrR

Crystals were obtained by using 1 μl of protein solution (concentration of 6.0 mg/ml) mixed with 1 μl of reservoir solution using the hanging-drop vapor diffusion method at 285 K. The reservoir solution contained PEG 20000 (11, 13, 15, 17 and 21% (w/v)) and 0.1 M MES pH (6.0, 6.5, 7.0 and 7.5). Crystals were obtained after three weeks and grew to their maximum dimensions within one month. Two different crystal forms, rectangular plate-shaped crystals and thin plates, were observed in the same drop. Both crystals forms were transferred into a buffer containing the reservoir solution plus 30% (v/v) ethylene glycol for 5 min prior to flash cooling using liquid nitrogen. For phasing, 20 mM tetra-chloro platinate IV (Hampton Research) was added to the crystallization drop, and the rectangular plate-shaped crystals were soaked for 30 min. The crystals with no obvious optical damage were harvested and flash-cooled in liquid nitrogen following the procedure above.

### Data collection

Initially crystals were screened for quality at beamline P13 (DESY, EMBL Hamburg). All X-ray diffraction data were collected at beamline ID23eh1 of the European Synchrotron Radiation Facility (ESRF), Grenoble [45]. All data sets were processed and scaled using XDS and XSCALE software package [46]. Data sets from both native crystal forms were collected at 100 K. A single-wavelength anomalous dispersion (SAD) dataset from a single heavy-atom derivatized crystal (rectangular plate-shaped crystal) was collected at 1.0714 Å at 100 K. Diffraction data up to 1.7 Å was used for heavy atom localization and subsequent phasing.

### Structure determination of NsrR

The structure of the thin plate-shaped crystals was solved by molecular replacement using the structure of the receiver domain of PhoB (PDB entry: 1B00) [47] as a model to phase the native data set at 1.8 Å resolution. The model generated was refined manually in COOT followed by iterative cycles of refinement using the program *phenix*.*refine* [48]. Manual adjustments between the refinement cycles were performed with the program COOT [49] and Ramachandran validation was done using MolProbity [50].

The SAD dataset of the rectangular plate-shaped crystal was used for phasing *via* the Auto-Rickshaw server [51]. The initial model was further built and refined manually using COOT [49] and *phenix*.*refine* from the Phenix package [48] with iterative cycles of refinement. This model was used to phase the native data set of the rectangular plate-shaped crystals at a resolution of 1.6 Å.

Data collection and refinement statistics are listed in Table 1 and all images of the models were prepared using PyMOL [52].

**Table 1:** Data collection, phasing, and refinement statistics for the receiver and effector domains of NsrR.

	NsrR-RD (native)	NsrR-ED (native)	NsrR-ED (SAD dataset)
**Data collection**			
Space group	P 2_1_ 2_1_ 2	P 2_1_ 2_1_ 2	P 2_1_ 2_1_ 2
*Cell dimensions*			
a, b, c (Å)	57.0 107.1 39.4	56.3 60.4 56.8	56.3 60.6 56.7
α, β, γ (°)	90.0 90.0 90.0	90.0 90.0 90.0	90.0 90.0 90.0
Wavelength (λ)	1.0688	0.9677	1.0714
Resolution (Å)	39.48–1.80 (1.86–1.80)	56.85–1.60 (1.65–1.60)	100.00–1.70 (1.75–1.70)
R_merge_ ^a^	3.4 (33.3)	4.8 (30.5)	6.8 (97.0)
I /σ(I)	26.2 (5.1)	18.2 (4.6)	21.6 (1.7)
Completeness (%)	98.8 (98.8)	99.5 (99.7)	98.8 (90.2)
Redundancy	4.8 (4.8)	4.8 (4.9)	11.7 (6.5)
**Structure Refinement**			
Resolution (Å)	39.48–1.80 (1.86–1.80)	56.85–1.60 (1.65–1.60)	
No. of reflections	109201 (10602)	124810 (12438)	
CC1/2	0.999 (0.924)	0.999 (0.923)	
R_work_^b^ / R_free_ ^b^	0.17 (0.20)/ 0.22 (0.27)	0.18 (0.22)/ 0.22 (0.27)	
*No*. *of atoms*	2027	1843	
Macromolecules	1894	1580	
Ligand/ion	20	8	
Water	113	255	
*B-factors (Å*^*2*^*)*	28.3	21.7	
Macromolecules	27.7	20.2	
Ligand/ion	34.0	38.6	
Solvent	36.9	30.4	
*R*.*m*.*s*. *deviations*			
Bond lengths (Å)	0.007	0.008	
Bond angles (°)	1.11	1.18	
*Ramachandran plot (%)*			
Favored	99.0	97.0	
Allowed	1.0	2.48	
Outliers	0.0	0.52	

Values in parentheses are for the highest resolution shell.

^a^ R_merge_ is defined as *R*_*sym*_ = ∑_*hkl*_∑_*i*_|*I*_*i*_(*hkl*) − ⟨*I*(*hkl*)⟩|/∑_*hkl*_∑_*i*_*I*_*i*_(*hkl*) and

^b^ R_F_ as *R*_*f*_ = ∑_*hkl*_‖*F*_*obs*_|−|*F*_*calc*_‖/∑_*hkl*_|*F*_*obs*_|

### Accession numbers

Coordinates and structure factors have been deposited in the PDB with accession numbers 5DCL (NsrR-RD) and 5DCM (NsrR-ED).

## Results and Discussion

**Fig 1:**
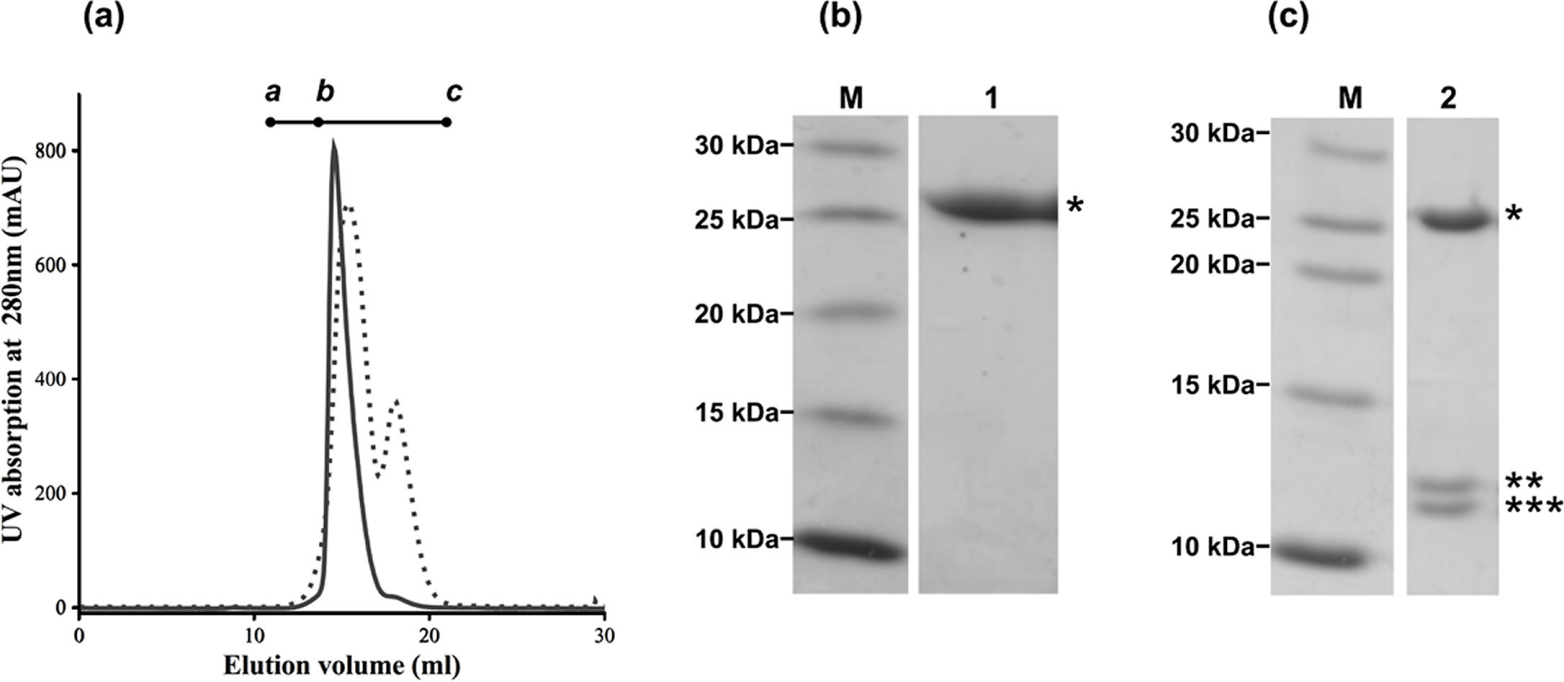
Purification of NsrR and SDS PAGE analysis of purified NsrR directly and one week after purification. (a) Elution profile of size-exclusion chromatography step of NsrR. The y-axis represents the UV absorption of the protein at 280 nm, while the x-axis represents the elution volume. a, b, c refer to the protein standards dextran blue (2,000 kDa), BSA (67 kDa), and lysozyme (14.3 kDa), respectively. The bold line represents the chromatogram of freshly purified NsrR while the dashed line shows the chromatogram of the same NsrR protein after one week. (b) Freshly purified NsrR protein, and (c) NsrR protein after one week. Lanes: M represents the PAGE Ruler Unstained Ladder; 1: NsrR after a two-step purification; 2: NsrR one week after purification. * corresponds to full-length NsrR protein at 27 kDa, while ** and *** correspond to the NsrR-RD and NsrR-ED domain at around 13 kDa, respectively.

NsrR was expressed and purified as described [43], resulting in a homogenous protein as observed by size exclusion chromatography (Fig 1A), with a yield of 2 mg per liter of cell culture. By calibrating the column with proteins of known molecular weight the NsrR full length protein elutes as a dimer. The purified NsrR protein has a theoretical molecular mass of 27.7 kDa and was >98% pure as assessed by SDS-PAGE (Fig 1B, indicated by *). Surprisingly, over time NsrR degraded into two distinct fragments as visible on SDS-PAGE analysis using the same purified protein sample after one week (Fig 1C, indicated by ** and ***, respectively). This was also observed by size exclusion chromatography where a peak at an elution time of 18 min appeared (Fig 1A). Both bands were subjected to mass spectrometry analysis. The analysis revealed that the larger fragment (**) represents the N-terminal receiver domain (residues 1–119; referred to as NsrR-RD) whereas the smaller fragment (***) contained the C-terminal DNA-binding effector domain of NsrR (residues 129–243 including 21 amino acids derived from the expression tag; referred to as NsrR-ED) (Fig 1C). Residues 120–128 form the linker connecting the RD and ED. Such a cleavage of the full-length RR into two specific domains is not unusual and has been previously reported for other RRs as well [53]. Mass spectrometry analysis did not reveal the presence of any specific protease in the purified NsrR sample. Furthermore, addition of a protease inhibitor, such as PMSF (Phenylmethylsulfonyl fluoride) and AEBSF {4-(2-Aminoethyl) benzenesulfonyl fluoride hydrochloride}, even at high concentrations, did not inhibit proteolysis (data not shown).

Since formation of the crystals took around one month, it is not surprising that this cleavage also occurred in the crystallization drop. NsrR was crystallized yielding two crystal forms, which were distinguishable by visual inspection. Initially, we tried to solve the structure of NsrR by molecular replacement, which was not successful. Therefore, we tried heavy atom phasing using a platinum compound. This succeeded for the rectangular plate-shaped crystals. After the structure was solved, it became evident that these crystals contained two monomers of the ED of NsrR in the asymmetric unit.

We also tried to solve the structure of the thin plate-shaped crystals with this template, but the resulting model generated was not sufficient. Therefore, we thought that these crystals contained the N-terminal domain of NsrR and successfully phased this dataset using molecular replacement with the N-terminal domain of PhoB (PDB code: 1B00; as a template. This approach revealed that this crystal form indeed contained two monomers of the RD of NsrR in the asymmetric unit. Since both crystals forms were obtained in the same drop it is not surprising that, when dissolving several crystals and performing subsequent mass-spectrometry to identify the protein in the crystals, it yielded peptide fragments throughout the NsrR sequence [43].

In summary, the two crystal forms contained one of the two domains, respectively, such that both domains were successfully crystallized. We determined the crystal structures of NsrR-RD and NsrR-ED separately. However, a part of the linker region (residues 120–128; _120_RRSQQFIQQ_128_; underlined are the amino acid residues not visible in either domain) could not be traced in the electron density.

### Overall structure of the N-terminal NsrR receiver domain (NsrR-RD)

The structure of the NsrR-RD was determined at a resolution of 1.8 Å (Table 1). The R_work_ and R_free_ values after refinement were 0.17 and 0.22, respectively. Ramachandran validation revealed that all residues (100%, 236 amino acids) were in the preferred or allowed regions [49]. The structure contained many ethylene glycol molecules arising from the cryo-protecting procedure. Data collection and refinement statistics are listed in Table 1.

The asymmetric unit contains two copies of NsrR-RD. Although the entire N-terminal receiver domain is composed of residues Met1-Leu119, only residues Asn4 to Arg121 of chain A (including residues Arg120 and Arg121 of the linker) and Gln5 to Ser122 of chain B (including residues Arg120 until Ser122 of the linker) could be traced in the electron density of NsrR-RD. For Asn85, Asp86, and Glu87 of chain A, poor electron density was observed for the side chains and, thus, these side chains were deleted during refinement and are not present in the final structure. Since the two monomers of NsrR-RD were virtually identical (rmsd of 0.6 Å over 116 Cα atoms for the two monomers). Therefore, the overall structure is described for monomer A only.

**Fig 2:**
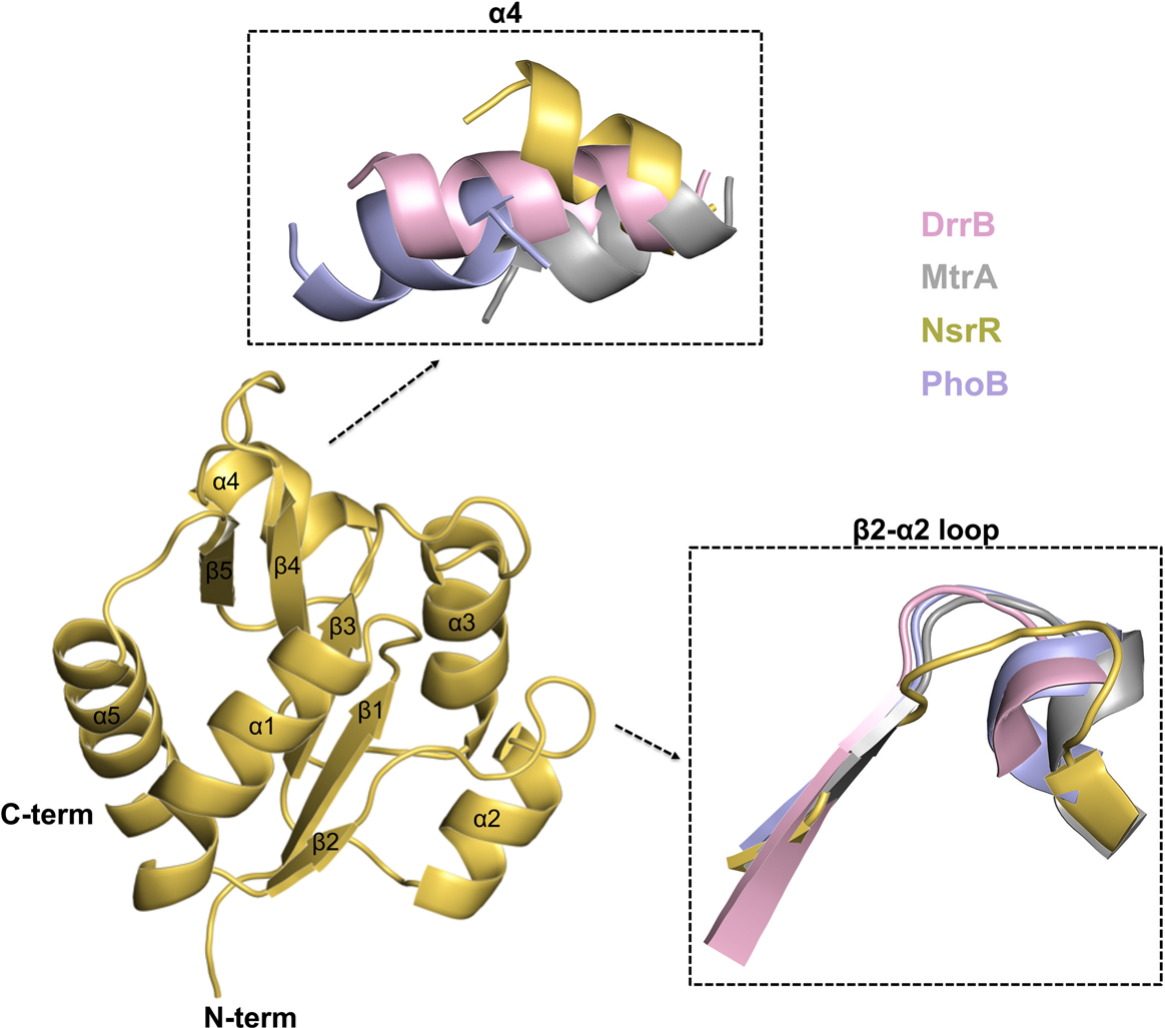
Structure of NsrR-RD. Cartoon representation of the helices (α1 – α5) and β-sheets (β1 - β5). Structural areas with the highest variations to the receiver domains of DrrB (pink, 1P2F), MtrA (grey, 2GWR), and PhoB (blue, 1B00) are marked in separate boxes.

NsrR-RD structurally adopts a αβ doubly-wound fold previously observed in OmpR/PhoB type regulators. Five β-strands (β1-β5) are arranged in a parallel fashion constituting the central core of the structure, which is surrounded by two α-helices (α1 and α5) on one and three helices (α2, α3, α4) on the other side (Fig 2). The NsrR-RD structure shows a β1-α1-β2-α2-β3-α3-β4-α4-β5-α5 topology as also observed for other RRs [40,41].

### Comparison with structures of other receiver domains

NsrR belongs to the OmpR/PhoB family of RRs. The receiver domain of NsrR was superimposed with other structurally characterized receiver domains from the OmpR/PhoB family, such as DrrB [40], KdpE [42], MtrA [37], and the crystal structure of only the receiver domain of PhoB [54]. The rmsd of the overlays and the corresponding PDB codes used are highlighted in Table 2. Superimposition of the structures revealed that helix α4 is slightly rotated outward in NsrR-RD (Fig 2). In receiver domains of response regulators, helix α4 has been shown to be a crucial part of the dimerization interface [55,56]. Furthermore, helix α4 in NsrR is shorter than in other RRs. The first helical turn is unwound and adopts an unstructured region (see Fig 2). A slightly outward rotation or unwinding of helix α4 has been observed in the structures of other RD of regulators. For example, the structure of BaeR [57] and RegX3 [36] displayed a completely unwound helix α4. In the structure of DrrD [41], helix α4 is only partially displaced. In the receiver domain of NsrR, helix α4 is also partially displaced but in a different direction (S1 **Fig**). Inspection of the crystal contacts revealed no major interactions in this region that could have influenced the orientation of helix α4. Furthermore, NsrR is crystallized as a monomer, and investigation of the symmetry-related molecules did not reveal a functional dimer within the crystal. This could explain the flexibility and thereby the different orientation of helix α4 in NsrR.

**Table 2:** The structures of the RD and ED domains of NsrR aligned to other response regulators. The rmsd values of the superimpositions of the structures of NsrR-RD and NsrR-ED with the available structures of members of the OmpR/PhoB subfamily are highlighted. *Seq ID (%) corresponds to the full-length protein sequence.

*Protein*	*PDB*	*Z-score*	*RMSD (Å)*	*Number of residues (total number of residues)*	*Seq*. ID (%)*	*Reference*
**Receiver domain**						
KdpE	4KNY	18.8	1.9	117 (222)	28	[42]
YycF	2ZWM	18.3	1.7	115 (120)	35	[53]
YycF	3F6P	18.1	1.7	114 (120)	35	[81]
DivK	1M5T	18.1	1.9	116 (123)	27	[65]
KdpE	1ZH2	18.0	1.9	115 (120)	28	[74]
PhoB	1B00	17.0	1.9	113 (122)	30	[47]
**Effector domain**						
PhoB	1GXQ	13.7	1.7	92 (105)	30	[54]
PhoP	2PMU	13.4	1.7	87 (93)	30	[82]
PhoB	2Z33	13.3	1.8	92 (104)	30	[83]
PhoB (DNA bound)	1GXP	13.3	2.0	92 (101)	30	[54]
SaeR	4IXA	13.0	2.1	94 (102)	29	Not available
RstA	4NHJ	11.8	1.9	85 (101)	29	[84]
KdpE	4KNY	11.5	2.6	86 (222)	28	[42]
**Full-length Response Regulators**						
	*PDB code*	*N-terminal rmsd (Å)*	*C-terminal rmsd (Å)*	*DNA bound*		*Reference*
DrrB	1P2F	2.1	2.3	No		[40]
DrrD	1KGS	2.1	1.9	No		[41]
KdpE	4KNY	1.9	2.6	Yes		[42]
MtrA	2GWR	2.1	2.0	No		[37]
PrrA	1YS6	2.0	2.2	No		[38]
RegX3	2OQR	2.3	2.1	No		[36]
BaeR	4B09	2.1	2.1	No		[57]
VraR	4GVP	2.3	2.6	No		[85]

Based on the Dali server [58], the NsrR-RD domain is structurally closely related to KdpE (PDB code: 4KNY) from *E*. *coli*, displaying a sequence identity of 28% [59]. This structural homology is also reflected by the low rmsd of 1.9 Å over 117 Cα atoms after superimposition of the receiver domains of NsrR and KdpE (Table 2) [42]. Furthermore, the orientation of the helix α4 in NsrR is close to that present in KdpE (S1 **Fig**).

### Active site residues and dimerization

**Fig 3:**
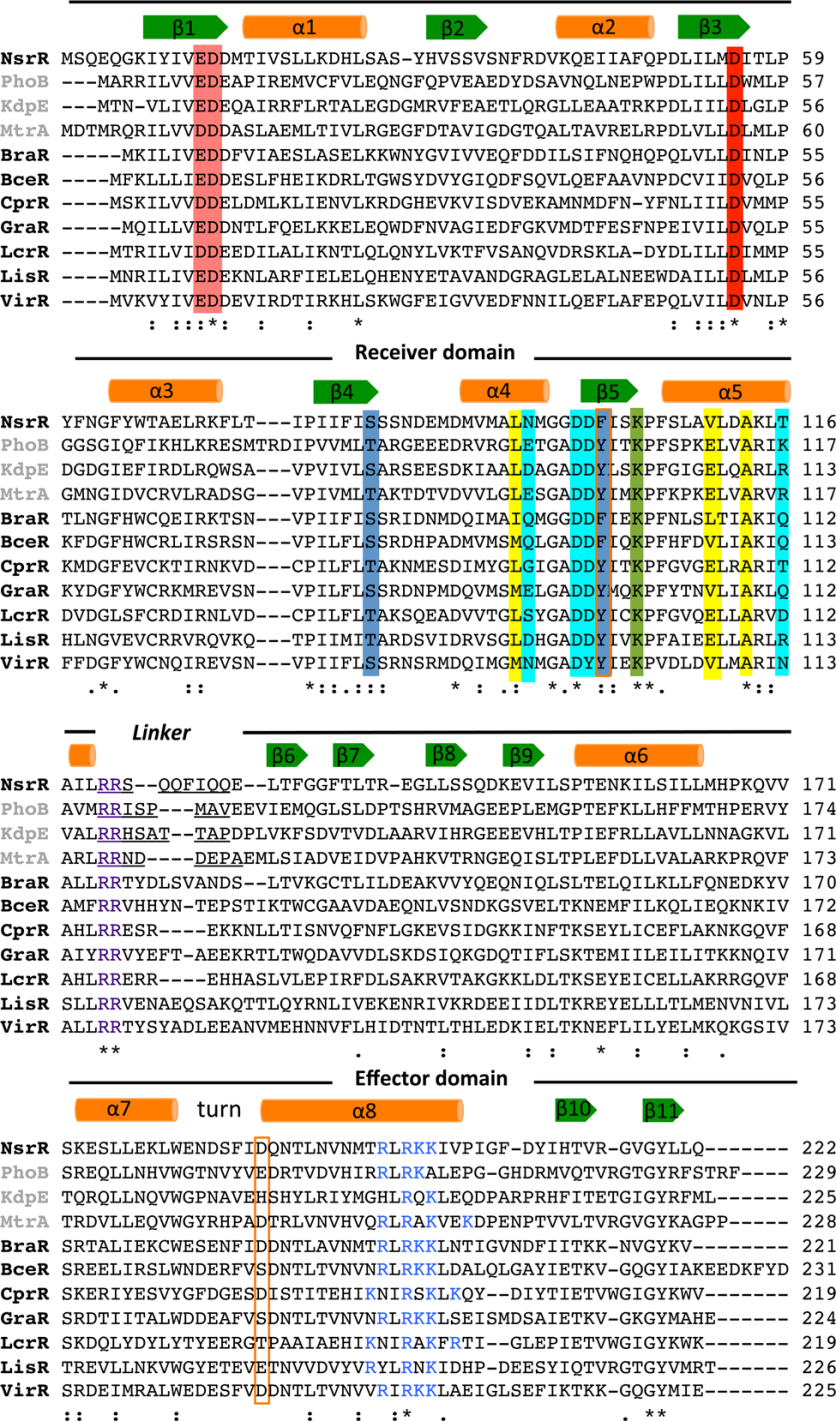
Sequence alignment of NsrR protein with other response regulators. A sequence alignment of NsrR with RRs belonging to the OmpR/PhoB subfamily (marked in grey) and RRs involved in lantibiotic resistance (black) is shown. The active site aspartate residue (highlighted in red), the residues forming the acidic pocket surrounding it (highlighted in pink), the switch residues (highlighted in blue), the conserved lysine residue (highlighted in green), the highly conserved residues of the linker region (colored in purple), the residues involved in dimer interface of receiver domain (highlighted in yellow), residues involved in interdomain interactions (shown in orange boxes and in cyan) and the residues involved in interaction with DNA (colored in blue) are shown. The linker region of the known structures is underlined within the sequence.

All RRs contain a highly conserved aspartate residue in the active site (Fig 3; shown in red). Phosphorylation of this aspartate residue induces a conformational change leading to the activation of the effector domain that binds DNA and regulates the transcription of target genes. This site of phosphorylation is conserved throughout the family of response regulators, including the lantibiotic resistance-associated RRs such as BraR from *L*. *monocytogenes* [26], BceR from *Bacillus subtilis* [27], CprR from *C*. *difficile* [60,61], GraR from *S*. *aureus* [62,63], LcrR from *S*. *mutans* [19], LisR [28], and VirR from *L*. *monocytogenes* [64] (Fig 3).

**Fig 4:**
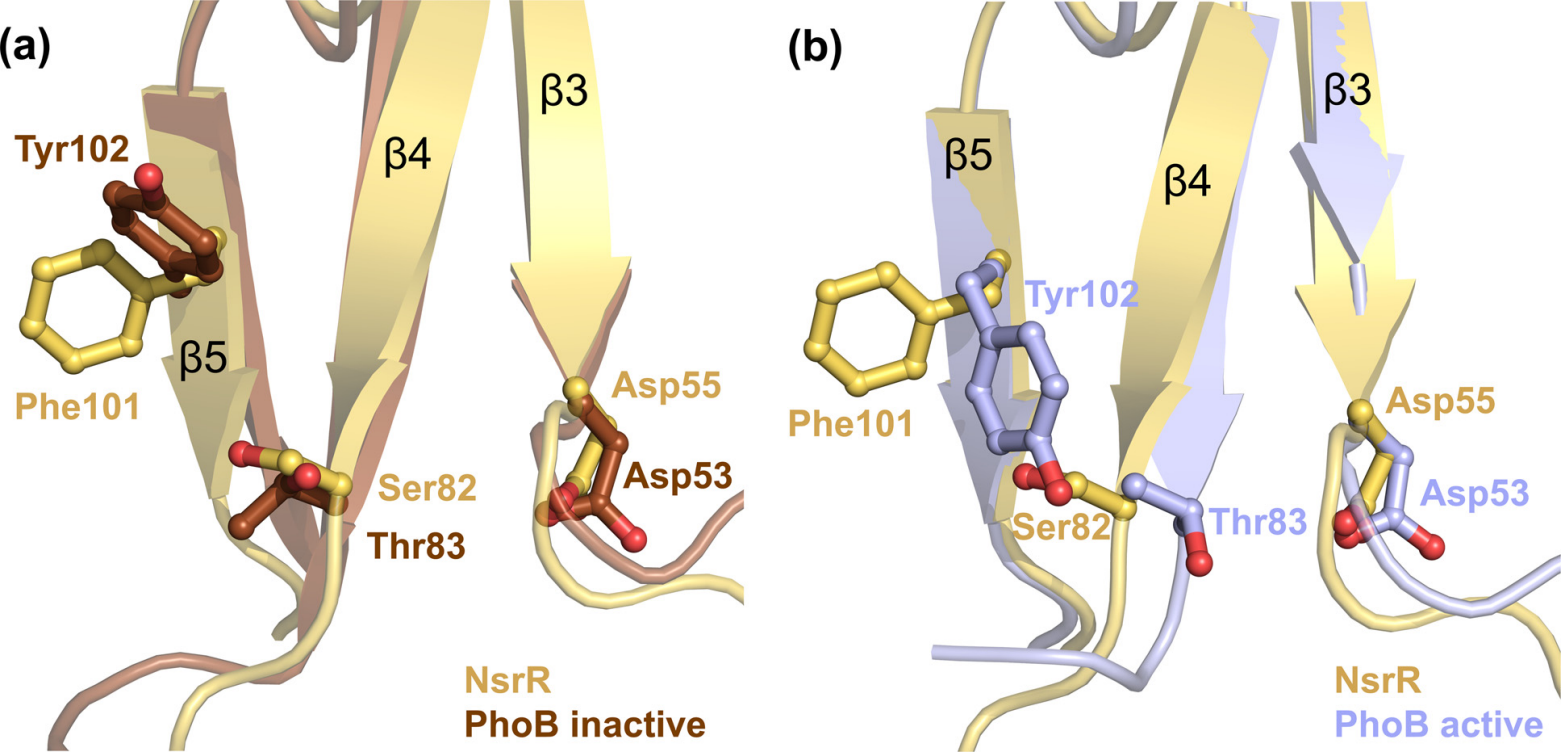
Location of the highly conserved Asp55 and inactive state conformation of the key switch residues, Ser82 and Phe101 in NsrR-RD. NsrR (represented in yellow) displays a geometry representing the inactive state as deduced from the inactive state structure of PhoB (shown in brown, PDB code 1B00) (a). The inactive conformation of NsrR differs from the active state structure of PhoB (light blue, PDB code 1ZES) (b) in the orientation of the corresponding switch residues, Ser82 and Phe101, which adopt a conformation pointing away from the active site (Asp55 in NsrR).

The putative phosphorylation site of NsrR is Asp55, which is localized at the end of strand β3 (Fig 3, shown in red; Fig 4) and lies within an acidic environment composed of the side chains of Glu12 and Asp13 (Fig 3, highlighted in pink). This pocket is similar to the acidic active site observed within most structures of RRs such as PhoB from *E*. *coli* [47], PhoP from *M*. *tuberculosis* [39], and DivK from *Caulobacter crescentus* [65]. In NsrR, Glu12, Asp13, and Asp55 are in close proximity of a highly conserved Lys104 residue (highlighted in green in Fig 3).

A divalent metal ion is usually bound in this acidic environment and is essential for phosphorylation and de-phosphorylation of RRs [66,67]. In some RRs like CheY, Mg^2+^ is observed in the structure, bound near the phosphorylation site [47,68,69]. In the KdpE regulator from *E*. *coli* that is involved in osmoregulation, a divalent calcium ion is present. However, the structure of NsrR-RD did not contain any divalent ion. Instead, a water molecule is present, which interacts with Glu12 of the acidic pocket, Lys104, and another water molecule in the vicinity.

Within the β4-α4 loop and in β5 of the RD of RRs, specific amino acids are crucial for signal transduction from the RD to the ED via conformational changes that are a consequence of phosphorylation of the RD. These amino acids are Ser/Thr and Phe/Tyr located at the end of β4 and before β5, respectively, and designated as “signature switch residues”. As seen in the alignment (Fig 3, highlighted in blue), these signature residues (Ser/Thr and Phe/Tyr) are highly conserved in the lantibiotic resistance-associated RRs. The orientation of the side chains of these residues determines whether the RD is in an active or inactive state [24,70]. In the inactive state, the phenylalanine or tyrosine residue faces away from the active site, and the corresponding serine or threonine residue adopts an outward-facing conformation as well [70–72] (Fig 4A). In contrast, the switch residues face towards the active site in the active state conformation (Fig 4B).

By sequence alignment with other lantibiotic resistance-associated RRs, these “signature switch residues” are identified as Ser82 and Phe101 in NsrR (see above). Although some RRs such as KdpE, BraR, BceR, GraR, and VirR contain a serine residue as the first switch residue, the others possess a threonine instead. Furthermore, the second switch residue is mostly a tyrosine, with NsrR, BraR, and BceR being the only exceptions containing a phenylalanine at that position. A comparison of the NsrR-RD structure with the available structures of PhoB (Fig 4) in the active (PDB code: 1ZES) and inactive (PDB code: 1B00) states demonstrates that Ser82 (NsrR-RD) is oriented away from the active site Asp55, and that Phe101 is also in an outward conformation suggesting an inactive state of the NsrR-RD (Fig 4A).

As mentioned above, RRs contain a phosphorylation-activated switch and normally exist in equilibrium between the active and inactive conformations. Phosphorylation shifts the equilibrium towards the active conformation [73] and induces the formation of rotationally symmetric dimers on the α4-β5-α5 interface of RDs [55]. It has been suggested that dimerization is crucial for DNA-binding of RRs of the OmpR/PhoB subfamily.

The RD domain of NsrR was crystallized with two separate monomers in the asymmetric unit. Therefore, we performed a DALI search [58] and focused on RD domains that were structurally determined as functional dimers. In this context, the dimer of full-length KdpE from *E*. *coli* (Z-score 18.8, rmsd 1.9 Å over 117 Cα atoms) (PDB code: 4KNY) [42] and the structure of the functional dimer of the RD of KdpE from *E*. *coli* (PDB code: 1ZH2) [74] represent the most structurally related structures.

We aligned NsrR-RD on both monomers of the RD of KdpE. Since helix α4 of NsrR-RD is orientated slightly different when compared with other structures of RDs (Fig 2), helix α4 and the N-terminal loop of one monomer were clashing with the second monomer (S2A **Fig**). Therefore, helix α4 and the N-terminal loop were shifted to the position of KdpE by primarily modifying backbone torsion angles in the region immediately C-terminal to helix α4. Afterwards, helix α4 and the adjacent loops were energy minimized with the MAB force field [75] as implemented in the program Moloc; all other atoms of NsrR-RD were kept fixed. The result is highlighted in S2B **Fig**. The energy minimized structure of NsrR-RD was then superimposed on the dimeric structure of KdpE [74].

**Fig 5:**
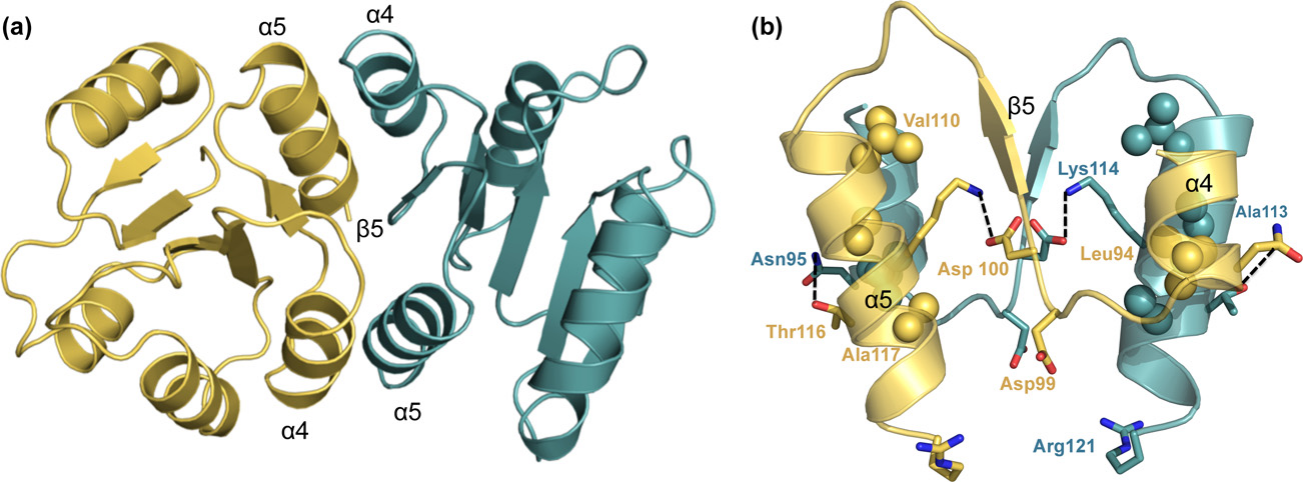
Functional dimer orientation of the RDs of NsrR. Dimeric structure of the RD of NsrR aligned to the structure of KdpE (PDB code 1ZH2, not shown). (a) The two monomers of NsrR as functional dimers are represented in a cartoon representation displayed in cyan and yellow colors. (b) Zoom-in of the dimeric interface mediated by α4-β5-α5. The monomer-monomer interactions are facilitated by hydrophobic residues (displayed as spheres), inter- and intra-domain interactions (displayed as sticks). The layout is adopted from [55].

The putative functional dimer of NsrR-RD is depicted in Fig 5. The dimeric interface is formed by α4-β5-α5 of RD (Fig 5A), as previously observed in other RRs [40,56,74]. In KdpE, a network of salt bridges and other electrostatic interactions stabilize the interface within a single monomer as well as between the monomers. Majority of these interactions involve residues that are highly conserved within the OmpR/PhoB subfamily of RRs. In addition, the dimeric interface of KdpE is characterized by hydrophobic patch formed by residues Ile88 (α4), Leu91 (α4), Ala110 (α5), and Val114 (α5). Structurally, a similar set of residues is also found in NsrR: Leu94 (α4), Val110 (α5) and Ala113 (α5), respectively (depicted as spheres in Fig 5B), which are conserved to some extent on sequence level (highlighted in yellow; Fig 3).

Conserved intermolecular electrostatic interactions further stabilize the monomer-monomer interaction of KdpE and are formed between Asp97 (β5) and Arg111 (α5), Asp96 (α4–β5 loop) and Arg118 (α5), and Asp92 (α4) and Arg113 (α5). Some of these interactions can also be identified in the dimeric model of NsrR-RD. Here, Asp100 (β5) and Lys114 (α5) form an interaction within one monomer, and an intermolecular interaction can be observed between Asn95 (α4) of one monomer with Thr116 (α5) of the other monomer (Fig 3, shown in cyan). Asp99 (α4–β5 loop; Fig 3, shown in cyan) points toward the side chain of Arg121. This interaction is also observed in KdpE (Asp96 (α4–β5 loop) and Arg118 (α5)). In KdpE, Arg111 is additionally stabilized by another intra-molecular salt bridge with Glu107 (α5). Interestingly, in NsrR-RD this amino acid corresponds to Val110 (highlighted in yellow in Fig 3). As observed in this alignment, the above-mentioned arginine residue (Arg111 in KdpE) is either an arginine or a lysine residue (Lys114 in NsrR) in all RRs used in the alignment (Fig 3, shown in cyan). Interestingly, whenever an arginine is present at this position (Arg111 in KdpE), a glutamate (Glu107 in KdpE) is present as well, presumably stabilizing the arginine side chain. However, when a lysine is present at this position, the glutamate is exchanged to a hydrophobic residue contributing to the hydrophobic patch described above. Additionally, it has been shown for PhoB from *E*. *coli* [76] and PhoP from *B*. *subtilis* [77] that mutating the corresponding residues involved in dimerisation (residues Asp100, Val110 and Lys114 in NsrR) results in monomeric form of response regulator which has lost the ability to dimerize as well as display reduced DNA binding capabilities.

### Overall Structure of C-terminal DNA-binding effector domain of NsrR

The structure of NsrR-ED from *S*. *agalactiae* was determined using experimental phases from a single-wavelength anomalous dispersion dataset from the rectangular plate-shaped crystal derivatized with platinum at a resolution of 1.6 Å in space group *P*2_1_2_1_2. The R_work_ and R_free_ values after refinement were 0.18 and 0.22, respectively. Ramachandran validation was done using MolProbity [50]. Almost all residues (99.48%, 193 amino acids) were in the preferred or allowed regions, while 0.52% (1 amino acid) were localized in the disallowed region. The latter is Glu128 (last residue of the linker region) of chain B that is involved in crystal contacts and, therefore, likely adopts an unfavorable conformation. The structure contained a few ethylene glycol molecules introduced by the cryo-protecting procedure. The data collection and refinement statistics are listed in Table 1.

The C-terminal effector DNA-binding domain of NsrR is about 13 kDa in size and consists of residues 129–243 (including 21 amino acid residues of the expression tag). Monomer A contains residue 129–224 and monomer B contain residues 128–225. For Asp147 of chain A and Glu174 of chain B, poor electron density was observed for the side chains and, thus, these side chains were removed during refinement. The asymmetric unit contains two copies of NsrR-ED related by two-fold rotational symmetry. An overlay revealed that both monomers display high similarity in their overall structure with an rmsd of 0.5 Å over 95 Cα atoms. We therefore describe for the overall structure only monomer A.

**Fig 6:**
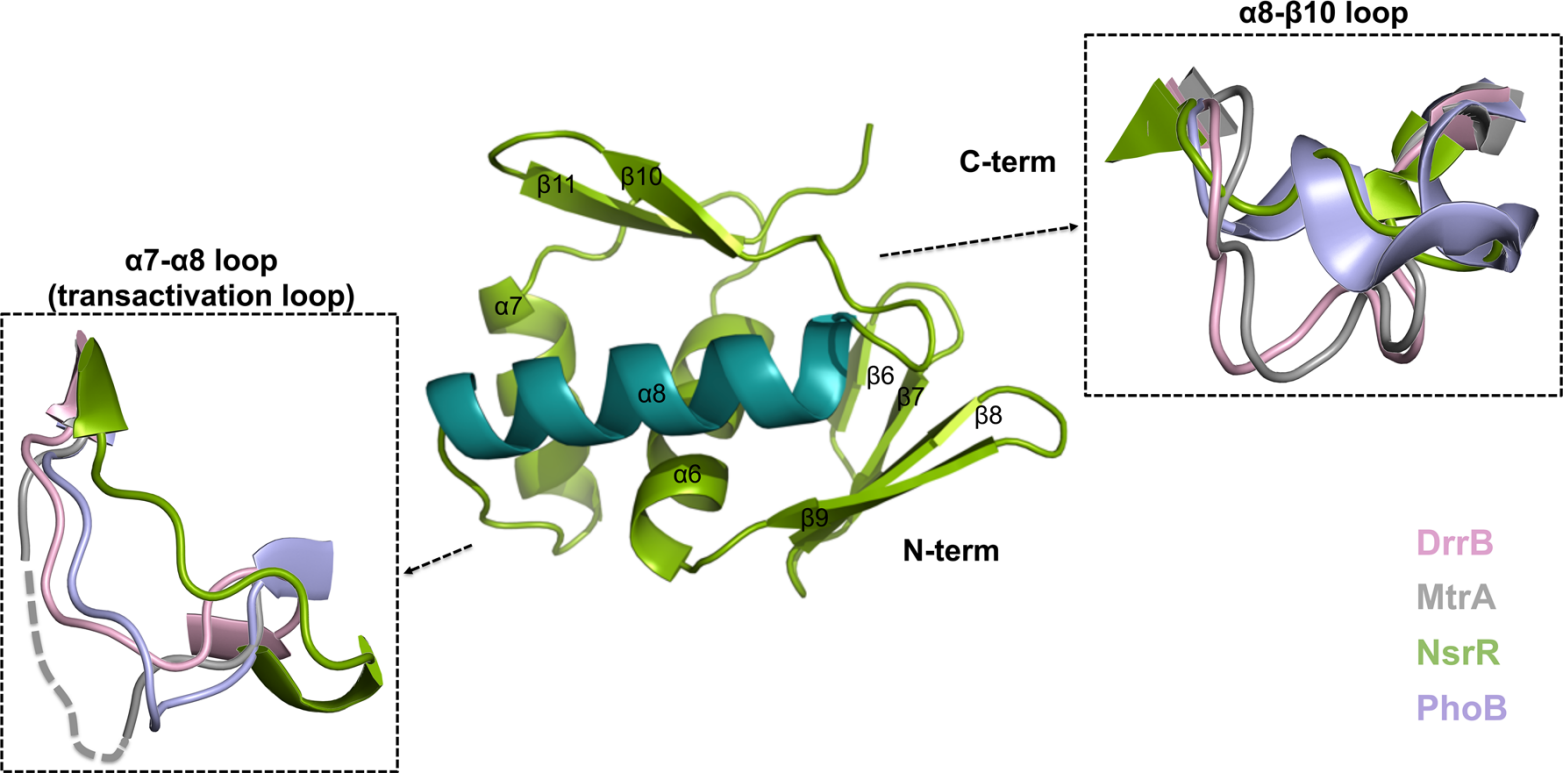
Structure of the C-terminal effector domain of NsrR. Cartoon representation of the C-terminal effector domain of NsrR (green; recognition helix in cyan). The structural areas with the highest variations compared to the effector domains of DrrB (pink, 1P2F), MtrA (grey, 2GWR), and PhoB (blue, 1GXQ) are marked. The transactivation loop of MtrA is missing in the structure, therefore, the two termini are connected by a dashed line.

The ED domain of NsrR consists of six β-strands and three α-helices in a β6-β7-β8-β9-α6-α7-α8-β10-β11 topology (the secondary structure elements are counted in continuation of those of the RD). The effector domain starts with a 4-stranded antiparallel β-sheet, followed by three α-helices and eventually ends in a C-terminal β-hairpin (Fig 6). The two β-sheets sandwich the three α-helices.

The characteristic feature of the OmpR/PhoB subfamily of RRs is a winged helix-turn-helix (wHTH) fold that is adopted by the α7-loop-α8 segment in full-length and single effector domain structures of RRs of this subfamily. The structure of NsrR-ED also contains such a wHTH motif built up by helices α7 and α8 (Fig 6). The second helix of the wHTH motif is important for DNA-binding and, therefore, is termed “recognition helix” [35] (shown in cyan in Fig 6). Furthermore, a helix within the HTH motif, named “positioning helix”, is important for proper orientation and positioning of the loop between these two helices and is referred to as “transactivation loop” (also called α-loop; Fig 6) [35,78]. In the structure of NsrR-ED, helix α8 is identified as the recognition helix, α7 as the positioning helix, and the loop region between helices α7-α8 as transactivation loop as observed in other RRs (Fig 6). The 16-residue long, solvent-exposed recognition helix α8 of NsrR-ED contains four positively charged residues that can potentially interact with DNA. These are Arg198, Arg200, Lys201, and Lys202. When comparing the sequence of NsrR with PhoB, KdpE, and MtrA, the alignment (Fig 3, colored in blue) emphasizes the variations at these positions, except for Arg200, which is conserved throughout the lantibiotic resistance RRs. Additionally, Lys202 is also highly conserved throughout the family of RRs except PhoB, clearly reflecting differences in the sequences of DNA to be bound.

### Comparison with structures of other effector domains

We performed a DALI search [58] to identify structurally related proteins to NsrR-ED. Here the structure of the effector domain of PhoB from *E*. *coli* (PDB code: 1GXQ) (Z-score of 13.7) [54] is structurally the most closely related. Similar to the PhoB effector domain, a 9-residues long loop (amino acid 182–189) is also present in the structure of NsrR-ED that connects helices α7 and α8. The rmsd between the three helices of the effector domain (including the two helices forming the wHTH motif) of PhoB [54] and NsrR-ED is 1.6 Å over 47 Cα atoms, clearly indicating that NsrR belongs to the OmpR/PhoB family of RRs.

Therefore, we superimposed the Cα traces of the effector domain of NsrR (NsrR-ED) with other previously determined effector domains from the OmpR/PhoB family such as DrrB [40], MtrA [37] and of only the effector domain structure of PhoB [54] from *E*. *coli*. Overall, the structures are very similar with rmsd’s ranging from 1.7 to 2.6 Å (Table 2). The highest variations (Fig 6) are visible in in the loop regions α7-α8, which corresponds to the transactivation loop. Interestingly, this region also shows low sequence conservation (Fig 3). In many RRs this transactivation loop along with the recognition helix α8, form inter-domain contacts in the inactive state and are only exposed upon activation of the RRs via a conformational change where the N- and C-terminal domains move away from each other [38].

### Linker region

The linkers that connect the RDs and EDs in response regulators are highly variable with respect to both length and sequence. The exact boundaries of these linkers are difficult to predict from sequence alignments in the absence of structural information of the distinct RR. Linker lengths in OmpR/PhoB proteins of unknown structure have been estimated by comparing the number of residues between conserved landmark residues in the regulatory and effector domains to those from structurally characterized family members. Such analysis has indicated that linker lengths vary from 5 to 20 residues [35]. Similar to the OmpR/PhoB family, the lantibiotic resistance-associated family of response regulators also displays diverse linker regions, which are recognized in sequence alignments by the introduction of gaps (Fig 3). Interestingly, two arginine residues (Arg120 and Arg121 in NsrR; Fig 3, shown in purple) at the end of the RDs seem to be strictly conserved throughout the family of response regulators in both the OmpR/PhoB and lantibiotic resistance-associated RRs, indicating a conserved similarity. As seen in the structures of MtrA and KdpE [37,74], this arginine residue residing at the end of α5 participates in the active state dimer interface of the RD through a salt bridge interaction [56] with an aspartate residue. This aspartate residue is identified in NsrR as Asp99 (see above). Arginine 121 of NsrR points towards this Asp99 residue however, the distance for a salt bridge interaction is too large.

Although we aimed at crystallizing full-length NsrR, this endeavor failed due to proteolytic cleavage within the linker region during the time period of crystallization. Nonetheless, the structures of NsrR-RD and NsrR-ED together provide the required structural knowledge to predict the linker region that joins the receiver and effector domains. The linker region of NsrR consists of approximately nine residues (Fig 3), comprising _120_RRSQQFIQQ_128_ (underlined residues are neither present in the structure of RD nor in ED of NsrR) and contains two positively charged amino acids.

### DNA-binding mode of NsrR using a full-length model

Since the structures of both domains of NsrR were determined, we used this structural information together with the available crystal structures of related proteins to create a model of the full-length NsrR in its active and inactive state.

To achieve this, we first carefully analyzed the outcome of the Dali search for each domain and identified structurally highly similar proteins (based on Z-scores and rmsd values) and choose the full-length structures previously reported. This resulted in a list of possible templates for modeling the full-length structure of NsrR (Table 2). In solution, RRs exist in equilibrium between the active and inactive state, which is shifted towards the active state upon phosphorylation of the ED. This results in oligomerization of the RR and a higher affinity towards DNA.

**Fig 7:**
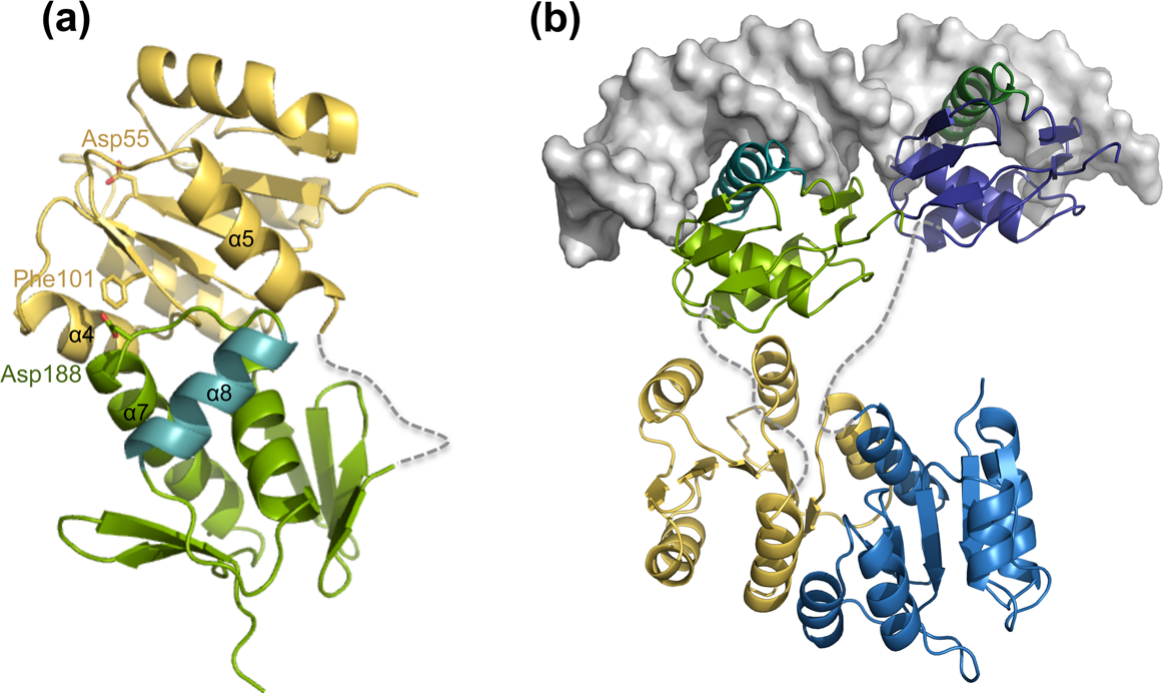
Model of full-length NsrR in its inactive state and active state. The RD domain of NsrR is highlighted in yellow and the ED domain in green with the “recognition helix” colored in cyan. (a) Inactive state conformation: Both domains of NsrR were aligned to the structure of MtrA (not shown), which adopts a closed inactive conformation, to obtain a model of full-length NsrR. Phe101 and Asp187 stabilize this closed conformation. The missing linker is represented by a dotted line. (b) Active state conformation: A model of full-length NsrR in active conformation based on the alignment of both the domains of NsrR to the structure of DNA bound structure of KdpE (PDB code: 4KNY), adopting an active open conformation, where the other molecule of NsrR is shown in shades of blue with the recognition helix colored in green.

Based on the above-mentioned criteria, the structure of MtrA from *M*. *tuberculosis*, crystallized in an inactive and non-phosphorylated state [37], seemed best suited for modeling purposes. Furthermore, the linker between the two domains of MtrA contains nine amino acids and is of similar length as the linker of NsrR. We aligned the NsrR-RD and -ED to the corresponding MtrA domains and evaluated the structure. This mimics the closed inactive conformation of NsrR (Fig 7A; the missing linker is represented as dotted line).

In MtrA, the two domains interact via the α4-β5-α5 interface of the receiver domain and the end of α7, α7-α8 loop and α8 of the effector domain. Both interfaces have been shown to form functionally important contact areas in the active state within members of the OmpR/PhoB subfamily. In our model of full-length NsrR, a similar orientation between the domains is observed, contributing to the inter-domain interactions. The inactive conformation of MtrA is supported by the orientation of the side chain of Tyr102, which points away from the active Asp56 residue, while the side chain of Tyr102 interacts with Asp190 of the RD of MtrA, thereby stabilizing its closed conformation. In the model of NsrR, similar amino acids are present, Phe101 (switch residue) and Asp188 (Fig 3, represented by orange boxes) forming a likewise similar network of interaction.

Next, we were interested in the active conformation of the NsrR protein adopting an active “open” conformation in the dimeric state. We compared and aligned the NsrR-RD and ED on the dimeric structure of KdpE that was solved in the DNA-bound state [42,79] (Fig 7B).

Also the linker region of KdpE is of similar length as of NsrR, which suggests that the distance in the DNA-bound state between the RD and ED of NsrR will be similar to that in the KdpE active dimer. We superimposed the ED of NsrR with the DNA-binding domain of KdpE resulting in a reasonably well-aligned structure (rmsd of 2.6Å over 86 Cα atoms; Table 2). As a result, a highly positive groove is created by the two ED domains of NsrR which likely represents the DNA binding site as observed in KdpE. A prediction of the putative promoter sequence that NsrR binds via the BPROM online server [80] was performed (S3 **Fig**). A promoter region was identified upstream of the *nsr* operon. However, the regulation of the predicted promoter and the DNA binding by NsrR has to be confirmed.

### Conclusion

In numerous pathogenic bacteria such as *S*. *agalactiae*, *S*. *aureus*, and *C*. *difficile* that apparently do not produce a lantibiotic, a gene cluster is present to provide resistance against lantibiotics such as nisin, nukacin ISK-1, lacticin 481 gallidermin, actagardine, or mersacidin [18,22,60]. The regulation of the expression of these genes is mediated by two-component systems. The structure of the response regulator NsrR from *S*. *agalactiae* presented in this study is the first structural information available for the subgroup of lantibiotic resistance-associated RRs.

## Supporting Information

S1 Fig Structural alignment of the helix α4 in structures of different response regulators. (DOCX)

S2 Fig The structure of the RD of NsrR is aligned with the corresponding domain of KdpE. (DOCX)

S3 Fig The predicted *nsr* promoter sequence. (DOCX)

## References

[1] Cotter PD, Ross RP, Hill C (2012) Bacteriocins—a viable alternative to antibiotics? Nature Reviews Microbiology 11: 95–105. 10.1038/nrmicro2937 23268227

[2] Bierbaum G, Szekat C, Josten M, Heidrich C, Kempter C, Jung G, et al (1996) Engineering of a novel thioether bridge and role of modified residues in the lantibiotic Pep5. Applied and environmental microbiology 62: 385–392. 8593044 10.1128/aem.62.2.385-392.1996 PMC167809

[3] Sahl H-G, Bierbaum G (1998) Lantibiotics: biosynthesis and biological activities of uniquely modified peptides from gram-positive bacteria. Annual Reviews in Microbiology 52: 41–79. 10.1146/annurev.micro.52.1.41 9891793

[4] Cotter PD, Hill C, Ross RP (2005) Bacterial lantibiotics: strategies to improve therapeutic potential. Current Protein and Peptide Science 6: 61–75. 15638769 10.2174/1389203053027584

[5] Dischinger J, Basi Chipalu S, Bierbaum G (2014) Lantibiotics: promising candidates for future applications in health care. International Journal of Medical Microbiology 304: 51–62. 10.1016/j.ijmm.2013.09.003 24210177

[6] Boakes S, Wadman S (2008) The therapeutic potential of lantibiotics. Innov Pharm Technol 27: 22–25.

[7] Alkhatib Z, Lagedroste M, Zaschke J, Wagner M, Abts A, Fey I, et al (2014) The C‐terminus of nisin is important for the ABC transporter NisFEG to confer immunity in *Lactococcus lactis*. MicrobiologyOpen 3: 752–763. 10.1002/mbo3.205 25176038 PMC4234265

[8] Wiedemann I, Breukink E, Van Kraaij C, Kuipers OP, Bierbaum G, De Kruijff B, et al (2001) Specific Binding of Nisin to the Peptidoglycan Precursor Lipid II Combines Pore Formation and Inhibition of Cell Wall Biosynthesis for Potent Antibiotic Activity. Journal of Biological Chemistry 276: 1772–1779. 11038353 10.1074/jbc.M006770200

[9] Engelke G, Gutowski-Eckel Z, Kiesau P, Siegers K, Hammelmann M, Entian K (1994) Regulation of nisin biosynthesis and immunity in *Lactococcus lactis* 6F3. Applied and environmental microbiology 60: 814–825. 8161176 10.1128/aem.60.3.814-825.1994 PMC201397

[10] Kuipers OP, Beerthuyzen MM, Siezen RJ, VOS WM (1993) Characterization of the nisin gene cluster nisABTCIPR of *Lactococcus lactis*. European Journal of Biochemistry 216: 281–291. 7689965 10.1111/j.1432-1033.1993.tb18143.x

[11] Guder A, Schmitter T, Wiedemann I, Sahl H-G, Bierbaum G (2002) Role of the single regulator MrsR1 and the two-component system MrsR2/K2 in the regulation of mersacidin production and immunity. Applied and environmental microbiology 68: 106–113. 11772616 10.1128/AEM.68.1.106-113.2002 PMC126572

[12] Alkhatib Z, Abts A, Mavaro A, Schmitt L, Smits SH (2012) Lantibiotics: how do producers become self-protected? Journal of biotechnology 159: 145–154. 10.1016/j.jbiotec.2012.01.032 22329892

[13] Chatterjee C, Paul M, Xie L, van der Donk WA (2005) Biosynthesis and mode of action of lantibiotics. Chemical Reviews 105: 633–684. 15700960 10.1021/cr030105v

[14] Saris PEJ, Immonen T, Reis M, Sahl H-G (1996) Immunity to lantibiotics. Antonie van Leeuwenhoek 69: 151–159. 8775975 10.1007/BF00399420

[15] Wolter AC, Duchardt-Ferner E, Nasiri AH, Hantke K, Wunderlich CH, Kreutz C, et al (2015) NMR resonance assignments for the class II GTP binding RNA aptamer in complex with GTP. Biomol NMR Assign. 10.1007/s12104-015-9646-7 26373429

[16] Christ NA, Bochmann S, Gottstein D, Duchardt-Ferner E, Hellmich UA, Dusterhus S, et al (2012) The First structure of a lantibiotic immunity protein, SpaI from *Bacillus subtilis*, reveals a novel fold. J Biol Chem 287: 35286–35298. 10.1074/jbc.M112.401620 22904324 PMC3471728

[17] Pozzi R, Coles M, Linke D, Kulik A, Nega M, Wohlleben W, et al (2015) Distinct mechanisms contribute to immunity in the lantibiotic NAI-107 producer strain *Microbispora* ATCC PTA-5024. Environ Microbiol. 10.1111/1462-2920.12892 25923468

[18] Khosa S, Alkhatib Z, Smits SH (2013) NSR from *Streptococcus agalactiae* confers resistance against nisin and is encoded by a conserved nsr operon. Biological chemistry 394: 1543–1549. 10.1515/hsz-2013-0167 23893686

[19] Kawada-Matsuo M, Oogai Y, Zendo T, Nagao J, Shibata Y, Yamashita Y, et al (2013) Involvement of the Novel Two-Component NsrRS and LcrRS Systems in Distinct Resistance Pathways against Nisin A and Nukacin ISK-1 in *Streptococcus mutans*. Applied and Environmental Microbiology 79: 4751–4755. 10.1128/AEM.00780-13 23709506 PMC3719517

[20] Falord M, Karimova G, Hiron A, Msadek T (2012) GraXSR proteins interact with the VraFG ABC transporter to form a five-component system required for cationic antimicrobial peptide sensing and resistance in *Staphylococcus aureus*. Antimicrobial agents and chemotherapy 56: 1047–1058. 10.1128/AAC.05054-11 22123691 PMC3264281

[21] Khosa S, Frieg B, Mulnaes D, Kleinschrodt D, Hoeppner A, Gohlke H, et al (2016) Structural basis of lantibiotic recognition by the nisin resistance protein from *Streptococcus agalactiae*. Sci Rep 6: 18679 10.1038/srep18679 26727488 PMC4698656

[22] Draper LA, Cotter PD, Hill C, Ross RP (2015) Lantibiotic Resistance. Microbiology and Molecular Biology Reviews 79: 171–191. 10.1128/MMBR.00051-14 25787977 PMC4394878

[23] Kleerebezem M, Quadri LE, Kuipers OP, De Vos WM (1997) Quorum sensing by peptide pheromones and two-component signal-transduction systems in Gram-positive bacteria. Molecular microbiology 24: 895–904. 9219998 10.1046/j.1365-2958.1997.4251782.x

[24] Stock AM, Robinson VL, Goudreau PN (2000) Two-component signal transduction. Annual review of biochemistry 69: 183–215. 10966457 10.1146/annurev.biochem.69.1.183

[25] Barrett JF, Hoch JA (1998) Two-component signal transduction as a target for microbial anti-infective therapy. Antimicrobial agents and chemotherapy 42: 1529–1536. 9660978 10.1128/aac.42.7.1529 PMC105640

[26] Hiron A, Falord M, Valle J, Débarbouillé M, Msadek T (2011) Bacitracin and nisin resistance in *Staphylococcus aureu*s: a novel pathway involving the BraS/BraR two-component system (SA2417/SA2418) and both the BraD/BraE and VraD/VraE ABC transporters. Molecular microbiology 81: 602–622. 10.1111/j.1365-2958.2011.07735.x 21696458

[27] Ohki R, Giyanto, Tateno K, Masuyama W, Moriya S, Kobayashi K, et al (2003) The BceRS two-component regulatory system induces expression of the bacitracin transporter, BceAB, in *Bacillus subtilis*. Molecular Microbiology 49: 1135–1144. 12890034 10.1046/j.1365-2958.2003.03653.x

[28] Cotter PD, Emerson N, Gahan CG, Hill C (1999) Identification and disruption of lisRK, a genetic locus encoding a two-component signal transduction system involved in stress tolerance and virulence in *Listeria monocytogenes*. Journal of bacteriology 181: 6840–6843. 10542190 10.1128/jb.181.21.6840-6843.1999 PMC94153

[29] Dintner S, Staroń A, Berchtold E, Petri T, Mascher T, Gebhard S (2011) Coevolution of ABC transporters and two-component regulatory systems as resistance modules against antimicrobial peptides in Firmicutes bacteria. Journal of bacteriology 193: 3851–3862. 10.1128/JB.05175-11 21665979 PMC3147537

[30] Mascher T (2006) Intramembrane-sensing histidine kinases: a new family of cell envelope stress sensors in Firmicutes bacteria. FEMS microbiology letters 264: 133–144. 17064367 10.1111/j.1574-6968.2006.00444.x

[31] Rietkötter E, Hoyer D, Mascher T (2008) Bacitracin sensing in Bacillus subtilis. Molecular microbiology 68: 768–785. 10.1111/j.1365-2958.2008.06194.x 18394148

[32] Staroń A, Finkeisen DE, Mascher T (2011) Peptide antibiotic sensing and detoxification modules of *Bacillus subtilis*. Antimicrobial agents and chemotherapy 55: 515–525. 10.1128/AAC.00352-10 21078927 PMC3028804

[33] Ouyang J, Tian X-L, Versey J, Wishart A, Li Y-H (2010) The BceABRS four-component system regulates the bacitracin-induced cell envelope stress response in *Streptococcus mutans*. Antimicrobial agents and chemotherapy 54: 3895–3906. 10.1128/AAC.01802-09 20606066 PMC2935011

[34] Gao R, Mack TR, Stock AM (2007) Bacterial response regulators: versatile regulatory strategies from common domains. Trends in biochemical sciences 32: 225–234. 17433693 10.1016/j.tibs.2007.03.002 PMC3655528

[35] Martínez-Hackert E, Stock AM (1997) Structural relationships in the OmpR family of winged-helix transcription factors. Journal of molecular biology 269: 301–312. 9199401 10.1006/jmbi.1997.1065

[36] King-Scott J, Nowak E, Mylonas E, Panjikar S, Roessle M, Svergun DI, et al (2007) The structure of a full-length response regulator from Mycobacterium tuberculosis in a stabilized three-dimensional domain-swapped, activated state. Journal of Biological Chemistry 282: 37717–37729. 17942407 10.1074/jbc.M705081200

[37] Friedland N, Mack TR, Yu M, Hung L-W, Terwilliger TC, Waldo GS, et al (2007) Domain orientation in the inactive response regulator Mycobacterium tuberculosis MtrA provides a barrier to activation. Biochemistry 46: 6733–6743. 17511470 10.1021/bi602546q PMC2528954

[38] Nowak E, Panjikar S, Konarev P, Svergun DI, Tucker PA (2006) The structural basis of signal transduction for the response regulator PrrA from *Mycobacterium tuberculosis*. Journal of Biological Chemistry 281: 9659–9666. 16434396 10.1074/jbc.M512004200

[39] Menon S, Wang S (2011) Structure of the response regulator PhoP from *Mycobacterium tuberculosis* reveals a dimer through the receiver domain. Biochemistry 50: 5948–5957. 10.1021/bi2005575 21634789 PMC3133661

[40] Robinson VL, Wu T, Stock AM (2003) Structural analysis of the domain interface in DrrB, a response regulator of the OmpR/PhoB subfamily. Journal of bacteriology 185: 4186–4194. 12837793 10.1128/JB.185.14.4186-4194.2003 PMC164896

[41] Buckler DR, Zhou Y, Stock AM (2002) Evidence of intradomain and interdomain flexibility in an OmpR/PhoB homolog from *Thermotoga maritima*. Structure 10: 153–164. 11839301 10.1016/s0969-2126(01)00706-7

[42] Narayanan A, Kumar S, Evrard AN, Paul LN, Yernool DA (2014) An asymmetric heterodomain interface stabilizes a response regulator–DNA complex. Nat Commun 5. 10.1038/ncomms4282 PMC4399498 24526190

[43] Khosa S, Hoeppner A, Kleinschrodt D, Smits SHJ (2015) Overexpression, purification and crystallization of the response regulator NsrR involved in nisin resistance. Acta Crystallographica Section F 71: 1322–1326. 10.1107/S2053230X15016441 PMC4601598 26457525

[44] Dyballa N, Metzger S (2009) Fast and sensitive colloidal coomassie G-250 staining for proteins in polyacrylamide gels. Journal of visualized experiments: JoVE. 10.3791/1431 PMC3149902 19684561

[45] Nurizzo D, Mairs T, Guijarro M, Rey V, Meyer J, Fajardo P, et al (2006) The ID23-1 structural biology beamline at the ESRF. Journal of synchrotron radiation 13: 227–238. 16645249 10.1107/S0909049506004341

[46] Kabsch W (2010) Xds. Acta Crystallogr D Biol Crystallogr 66: 125–132. 10.1107/S0907444909047337 20124692 PMC2815665

[47] Solà M, Gomis-RuÈth FX, Serrano L, González A, Coll M (1999) Three-dimensional crystal structure of the transcription factor PhoB receiver domain. Journal of molecular biology 285: 675–687. 9878437 10.1006/jmbi.1998.2326

[48] Adams PD, Afonine PV, Bunkóczi G, Chen VB, Davis IW, Echols N, et al (2010) PHENIX: a comprehensive Python-based system for macromolecular structure solution. Acta Crystallogr D Biol Crystallogr 66: 213–221. 10.1107/S0907444909052925 20124702 PMC2815670

[49] Emsley P, Lohkamp B, Scott WG, Cowtan K (2010) Features and development of Coot. Acta Crystallographica Section D: Biological Crystallography 66: 486–501. 20383002 10.1107/S0907444910007493 PMC2852313

[50] Chen VB, Arendall WB 3rd, Headd JJ, Keedy DA, Immormino RM, Kapral GJ, et al (2010) MolProbity: all-atom structure validation for macromolecular crystallography. Acta Crystallogr D Biol Crystallogr 66: 12–21. 10.1107/S0907444909042073 20057044 PMC2803126

[51] Panjikar S, Parthasarathy V, Lamzin VS, Weiss MS, Tucker PA (2009) On the combination of molecular replacement and single-wavelength anomalous diffraction phasing for automated structure determination. Acta Crystallogr D Biol Crystallogr 65: 1089–1097. 10.1107/S0907444909029643 19770506 PMC2756167

[52] Delano WL (2002) The PyMOL molecular graphics system.

[53] Okajima T, Doi A, Okada A, Gotoh Y, Tanizawa K, Utsumi R (2008) Response regulator YycF essential for bacterial growth: X-ray crystal structure of the DNA-binding domain and its PhoB-like DNA recognition motif. FEBS letters 582: 3434–3438. 10.1016/j.febslet.2008.09.007 18789936

[54] Blanco AG, Sola M, Gomis-Rüth FX, Coll M (2002) Tandem DNA Recognition by PhoB, a Two-Component Signal Transduction Transcriptional Activator. Structure 10: 701–713. 12015152 10.1016/s0969-2126(02)00761-x

[55] Toro-Roman A, Mack TR, Stock AM (2005) Structural analysis and solution studies of the activated regulatory domain of the response regulator ArcA: a symmetric dimer mediated by the α4-β5-α5 face. Journal of molecular biology 349: 11–26. 15876365 10.1016/j.jmb.2005.03.059 PMC3690759

[56] Bachhawat P, Swapna G, Montelione GT, Stock AM (2005) Mechanism of activation for transcription factor PhoB suggested by different modes of dimerization in the inactive and active states. Structure 13: 1353–1363. 16154092 10.1016/j.str.2005.06.006 PMC3685586

[57] Choudhury HG, Beis K (2013) The dimeric form of the unphosphorylated response regulator BaeR. Protein Science 22: 1287–1293. 10.1002/pro.2311 23868292 PMC3776340

[58] Holm L, Rosenström P (2010) Dali server: conservation mapping in 3D. Nucleic acids research 38: W545–W549. 10.1093/nar/gkq366 20457744 PMC2896194

[59] Altschul SF, Gish W, Miller W, Myers EW, Lipman DJ (1990) Basic local alignment search tool. Journal of molecular biology 215: 403–410. 2231712 10.1016/S0022-2836(05)80360-2

[60] Suárez JM, Edwards AN, McBride SM (2013) The Clostridium difficile cpr locus is regulated by a noncontiguous two-component system in response to type A and B lantibiotics. Journal of bacteriology 195: 2621–2631. 10.1128/JB.00166-13 23543720 PMC3676062

[61] McBride SM, Sonenshein AL (2011) Identification of a genetic locus responsible for antimicrobial peptide resistance in *Clostridium difficile*. Infection and immunity 79: 167–176. 10.1128/IAI.00731-10 20974818 PMC3019887

[62] Neoh H-m, Cui L, Yuzawa H, Takeuchi F, Matsuo M, Hiramatsu K (2008) Mutated response regulator graR is responsible for phenotypic conversion of *Staphylococcus aureus* from heterogeneous vancomycin-intermediate resistance to vancomycin-intermediate resistance. Antimicrobial agents and chemotherapy 52: 45–53. 17954695 10.1128/AAC.00534-07 PMC2223914

[63] Meehl M, Herbert S, Götz F, Cheung A (2007) Interaction of the GraRS two-component system with the VraFG ABC transporter to support vancomycin-intermediate resistance in *Staphylococcus aureus*. Antimicrobial agents and chemotherapy 51: 2679–2689. 17502406 10.1128/AAC.00209-07 PMC1932546

[64] Mandin P, Fsihi H, Dussurget O, Vergassola M, Milohanic E, Toledo Arana A, et al (2005) VirR, a response regulator critical for *Listeria monocytogene*s virulence. Molecular microbiology 57: 1367–1380. 16102006 10.1111/j.1365-2958.2005.04776.x

[65] Guillet V, Ohta N, Cabantous S, Newton A, Samama J-P (2002) Crystallographic and biochemical studies of DivK reveal novel features of an essential response regulator in *Caulobacter crescentus*. Journal of Biological Chemistry 277: 42003–42010. 12176983 10.1074/jbc.M204789200

[66] Lukat GS, Stock JB (1993) Response regulation in bacterial chemotaxis. Journal of cellular biochemistry 51: 41–46. 8381790 10.1002/jcb.240510109

[67] McCleary WR, Stock JB (1994) Acetyl phosphate and the activation of two-component response regulators. Journal of Biological Chemistry 269: 31567–31572. 7989325

[68] Stock AM, Martinez-Hackert E, Rasmussen BF, West AH, Stock JB, Ringe D, et al (1993) Structure of the magnesium-bound form of CheY and mechanism of phosphoryl transfer in bacterial chemotaxis. Biochemistry 32: 13375–13380. 8257674 10.1021/bi00212a001

[69] Bellsolell Ls, Cronet P, Majolero M, Serrano L, Coll M (1996) The three-dimensional structure of two mutants of the signal transduction protein CheY suggest its molecular activation mechanism. Journal of molecular biology 257: 116–128. 8632450 10.1006/jmbi.1996.0151

[70] West AH, Stock AM (2001) Histidine kinases and response regulator proteins in two-component signaling systems. Trends in biochemical sciences 26: 369–376. 11406410 10.1016/s0968-0004(01)01852-7

[71] Zhu X, Rebello J, Matsumura P, Volz K (1997) Crystal Structures of CheY Mutants Y106W and T87I/Y106W CheY Activation Correlates with Movement of Residue 106. Journal of Biological Chemistry 272: 5000–5006. 9030562 10.1074/jbc.272.8.5000

[72] Appleby JL, Bourret RB (1998) Proposed signal transduction role for conserved CheY residue Thr87, a member of the response regulator active-site quintet. Journal of bacteriology 180: 3563–3569. 9657998 10.1128/jb.180.14.3563-3569.1998 PMC107323

[73] Gardino AK, Kern D (2007) Functional Dynamics of Response Regulators Using NMR Relaxation Techniques. Methods in enzymology 423: 149–165. 17609130 10.1016/S0076-6879(07)23006-X

[74] Toro Roman A, Wu T, Stock AM (2005) A common dimerization interface in bacterial response regulators KdpE and TorR. Protein science 14: 3077–3088. 16322582 10.1110/ps.051722805 PMC2253231

[75] Gerber PR, Müller K (1995) MAB, a generally applicable molecular force field for structure modelling in medicinal chemistry. Journal of computer-aided molecular design 9: 251–268. 7561977 10.1007/BF00124456

[76] Mack TR, Gao R, Stock AM (2009) Probing the roles of the two different dimers mediated by the receiver domain of the response regulator PhoB. Journal of molecular biology 389: 349–364. 10.1016/j.jmb.2009.04.014 19371748 PMC2716121

[77] Chen Y, Birck C, Samama J-P, Hulett FM (2003) Residue R113 is essential for PhoP dimerization and function: a residue buried in the asymmetric PhoP dimer interface determined in the PhoPN three-dimensional crystal structure. Journal of bacteriology 185: 262–273. 12486063 10.1128/JB.185.1.262-273.2003 PMC141829

[78] Martínez-Hackert E, Stock AM (1997) The DNA-binding domain of OmpR: crystal structures of a winged helix transcription factor. Structure 5: 109–124. 9016718 10.1016/s0969-2126(97)00170-6

[79] Hasegawa H, Holm L (2009) Advances and pitfalls of protein structural alignment. Current opinion in structural biology 19: 341–348. 10.1016/j.sbi.2009.04.003 19481444

[80] Solovyev V, Salamov A (2011) Automatic annotation of microbial genomes and metagenomic sequences. Metagenomics and its applications in agriculture, biomedicine and environmental studies: 61–78.

[81] Zhao H, Heroux A, Sequeira RD, Tang L (2009) Preliminary crystallographic studies of the regulatory domain of response regulator YycF from an essential two-component signal transduction system. Acta Crystallographica Section F: Structural Biology and Crystallization Communications 65: 719–722. 10.1107/S1744309109022696 19574649 PMC2705644

[82] Wang S, Engohang-Ndong J, Smith I (2007) Structure of the DNA-binding domain of the response regulator PhoP from Mycobacterium tuberculosis. Biochemistry 46: 14751–14761. 18052041 10.1021/bi700970a PMC2535579

[83] Yamane T, Okamura H, Ikeguchi M, Nishimura Y, Kidera A (2008) Water-mediated interactions between DNA and PhoB DNA-binding/transactivation domain: NMR-restrained molecular dynamics in explicit water environment. Proteins: Structure, Function, and Bioinformatics 71: 1970–1983. 10.1002/prot.21874 18186481

[84] Li Y-C, Chang C-k, Chang C-F, Cheng Y-H, Fang P-J, Yu T, et al (2014) Structural dynamics of the two-component response regulator RstA in recognition of promoter DNA element. Nucleic acids research: gku572. 10.1093/nar/gku572 PMC4117788 24990372

[85] Leonard PG, Golemi-Kotra D, Stock AM (2013) Phosphorylation-dependent conformational changes and domain rearrangements in *Staphylococcus aureus* VraR activation. Proceedings of the National Academy of Sciences 110: 8525–8530. 10.1073/pnas.1302819110 PMC3666669 23650349

