# Supplementary material for: Structure of the Response Regulator NsrR from Streptococcus agalactiae, Which Is Involved in Lantibiotic Resistance
Source: PLoS One. 2016 Mar 1;11(3):e0149903. doi: 10.1371/journal.pone.0149903 (PMC4773095; doi:10.1371/journal.pone.0149903)

Supplementary Information:

**Structure of the Response Regulator NsrR From *Streptococcus agalactiae*, which is Involved in Lantibiotic Resistance**

**Sakshi Khosa*^1^,* Astrid Hoeppner*^2^_,_* Holger Gohlke*^3^*_,_ Lutz Schmitt*^1^*, and
Sander H.J. Smits*^1^****

*^1^*Institute of Biochemistry, Heinrich Heine University Duesseldorf, Universitaetsstr. 1, 40225 Duesseldorf, Germany

*^2^*X-Ray Facility and Crystal Farm, Heinrich Heine University Duesseldorf, Universitaetsstr. 1, 40225 Duesseldorf, Germany

*^3^*Institute of Pharmaceutical and Medicinal Chemistry, Heinrich Heine University Duesseldorf, Universitaetsstr. 1, 40225 Duesseldorf, Germany

******* Correspondence:

E-Mail: sander.smits@hhu.de

Phone: +49-211-81-12647

Fax: +49-211-81-15310

**Fig. S1:** **Structural alignment of the helix α4 in structures of different response regulators.**

(a) Structural comparison of the helix α4 of the RD domain of NsrR (green) with the helix α4 of BaeR (cyan), DrrD (pink), RegX3 (blue), and KdpE (yellow); (b) at 45° rotation; (c) zoom –in comparison of helix α4.


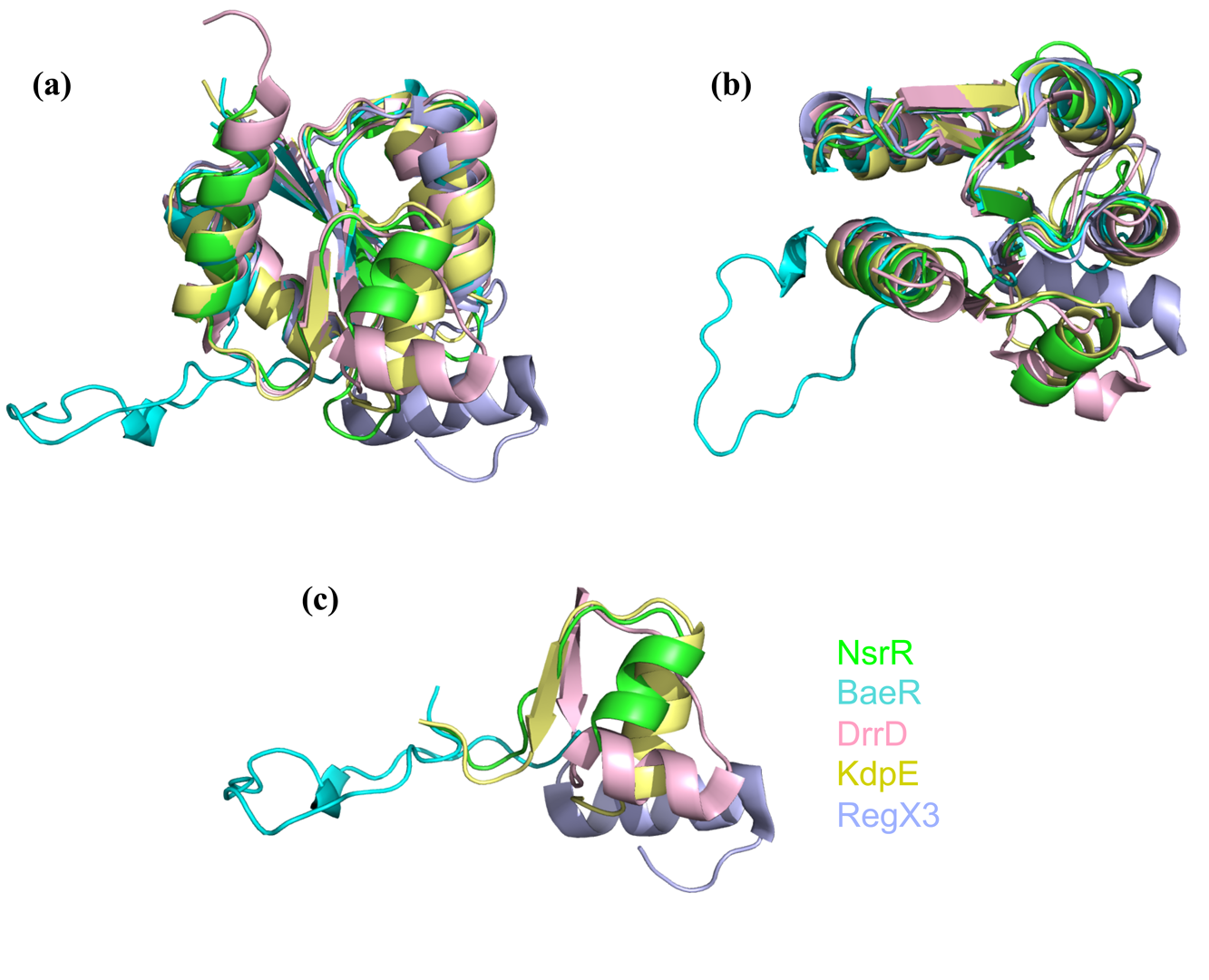

Supplement: S1 Fig — (DOCX) [file pone.0149903.s001.docx]
