# Supplementary material for: Structure of the Response Regulator NsrR from Streptococcus agalactiae, Which Is Involved in Lantibiotic Resistance
Source: PLoS One. 2016 Mar 1;11(3):e0149903. doi: 10.1371/journal.pone.0149903 (PMC4773095; doi:10.1371/journal.pone.0149903)

**Fig. S2:** **The structure of the RD of NsrR is aligned with the corresponding domain of KdpE.**

Structural comparison of the RD domain of NsrR with the receiver domain of KdpE (colored in silver) is shown.

(a) Comparison of helices α4 clearly indicates its slightly outward orientation in NsrR (shown in yellow).

(b) Alignment after energy minimization of the Cα atoms of helix α4 of NsrR (shown in brown).


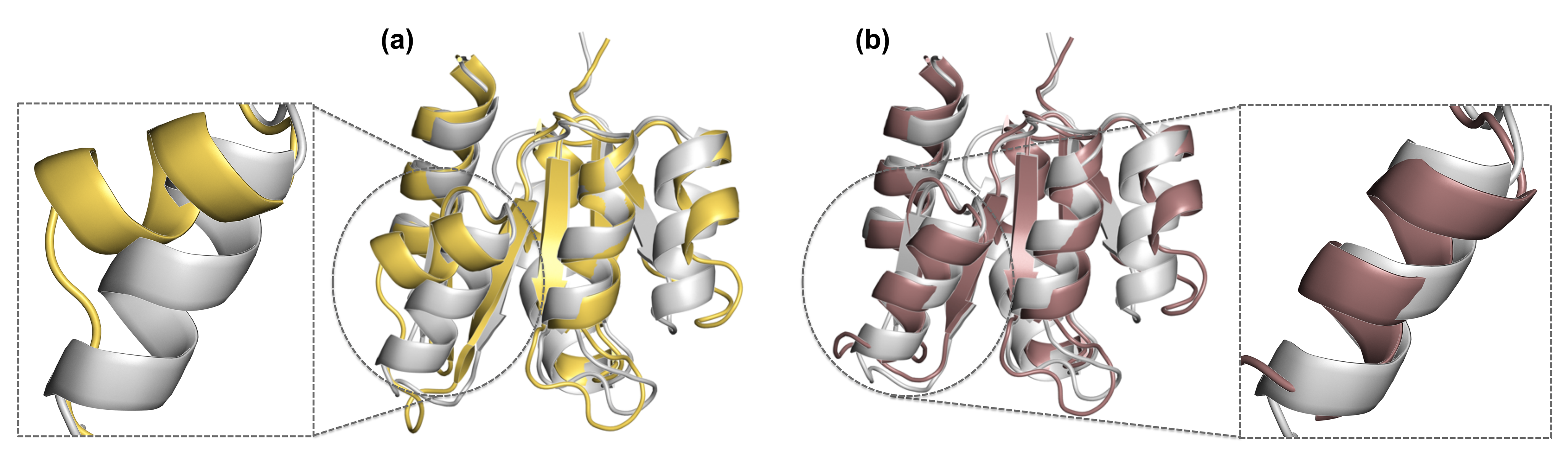

Supplement: S2 Fig — (DOCX) [file pone.0149903.s002.docx]
