# Supplementary material for: Structure of the Response Regulator NsrR from Streptococcus agalactiae, Which Is Involved in Lantibiotic Resistance
Source: PLoS One. 2016 Mar 1;11(3):e0149903. doi: 10.1371/journal.pone.0149903 (PMC4773095; doi:10.1371/journal.pone.0149903)

**Fig. S3: The predicted *nsr* promoter sequence.**

The main start site of the *nsr* promoter is shown with an arrow. The -35 and -10 sites are underlined while the start codon for the *nsr* gene is marked with an asterisk. The promoter prediction was carried out using BPROM (73).


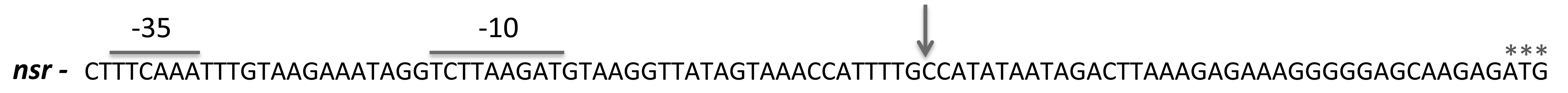

Supplement: S3 Fig — (DOCX) [file pone.0149903.s003.docx]
